# Long Chain Fatty Acid Esters of Quercetin-3-*O*-glucoside Attenuate H_2_O_2_-induced Acute Cytotoxicity in Human Lung Fibroblasts and Primary Hepatocytes

**DOI:** 10.3390/molecules21040452

**Published:** 2016-04-06

**Authors:** Sumudu N. Warnakulasuriya, Ziaullah, H. P. Vasantha Rupasinghe

**Affiliations:** 1 Department of Environmental Sciences, Faculty of Agriculture, Dalhousie University, Truro, NS B2N 5E3, Canada; sumudunw@dal.ca (S.N.W.); ziachem_315@yahoo.com (Z.); 2 Department of Pathology, Faculty of Medicine, Dalhousie University, Truro, NS B2N 5E3, Canada

**Keywords:** natural product derivatives, apoptosis, cytoprotection, human lung fibroblasts, human primary hepatocytes, lipid oxidation, oxidative stress

## Abstract

Cellular oxidative stress causes detrimental effects to macromolecules, such as lipids, nucleic acids and proteins, leading to many pathological conditions. Quercetin-3-*O*-glucoside (Q3G), a glycosylated derivative of quercetin (Q), is a natural polyphenolic compound known to possess antioxidant activity. The hydrophilic/lipophilic nature of an antioxidant molecule is considered as an important factor governing the accessibility to the active sites of oxidative damages *in vivo*. Six long chain fatty acid esters of Q3G were evaluated with comparison to Q and Q3G, for their cytoprotective activity under H_2_O_2_-induced oxidative stress using cell culture model systems through cell viability, lipid peroxidation and fluorescence microscopy studies. Pre-incubation of α-linolenic acid (ALA), eicosapentaenoic acid (EPA) and docosahexaenoic acid (DHA) esters of Q3G exhibited significantly (*p* ≤ 0.05) greater cell viability in both human lung fibroblast (WI-38) and human primary hepatocytes upon exposure to H_2_O_2_ insult when compared to the control. Cytoprotection due to oleic acid and linoleic acid esters of Q3G was observed only in human primary hepatocytes. All the derivatives, Q3G and quercetin showed ability to significantly (*p* ≤ 0.05) lower production of lipid hydroperoxides under induced oxidative stress, compared to the control. However, ALA and DHA esters of Q3G resulted in significantly lower lipid hydroperoxidation than Q and Q3G. Based on fluorescence microscopy study, H_2_O_2_-induced apoptosis was attenuated by the fatty acid derivatives of Q3G. The fatty acid derivatives of Q3G possess better cytoprotective effect than Q3G against H_2_O_2_-induced cytotoxicity *in vitro* and the concentration should be selected to avoid cytotoxicity.

## 1. Introduction

In biological environments, an excess production of partially reduced forms of molecular oxygen and insufficient antioxidant defense system disturbs the cellular redox balance resulting in oxidative stress [1] which is associated with many pathological conditions, such as inflammation, aging, cancer, atherosclerosis, hypertension and diabetes [2]. As a result of anthropogenic activities, a large number of organic and inorganic pollutants are released to the environment causing the generation of elevated levels of reactive oxygen species (ROS) in the human body. In addition to endogenous production of ROS, exposure to exogenous factors causes excess oxidative stress which cannot be counteracted by the antioxidant defense mechanisms in the target organs [3,4]. Liver is the main target for the toxic effects of xenobiotics resulting in oxidative stress which is involved in initiation and progression of pathogenesis of hepatic damage [5,6]. The lungs are a highly vascularized organ with a large surface area exposed to air and therefore, it is associated with higher oxygen concentrations than other organs. Consequently, lung is highly subjected to both exogenous and endogenous oxidative stress resulting in the pathogenesis of a number of chronic obstructive airway diseases, such as bronchitis and emphysema [7,8,9,10].

Therefore, dietary antioxidants are needed to minimize oxidative damage and recently, there is a global trend for using natural products as antioxidant supplements [2,11]. Antioxidant activity of natural polyphenolic compounds was reported to provide cytoprotective effect under induced oxidative stress *in vitro* and *in vivo* [11]. The lipophilic/hydrophilic nature of antioxidants is a crucial factor which limits their cellular uptake [12].

**Figure 1:**
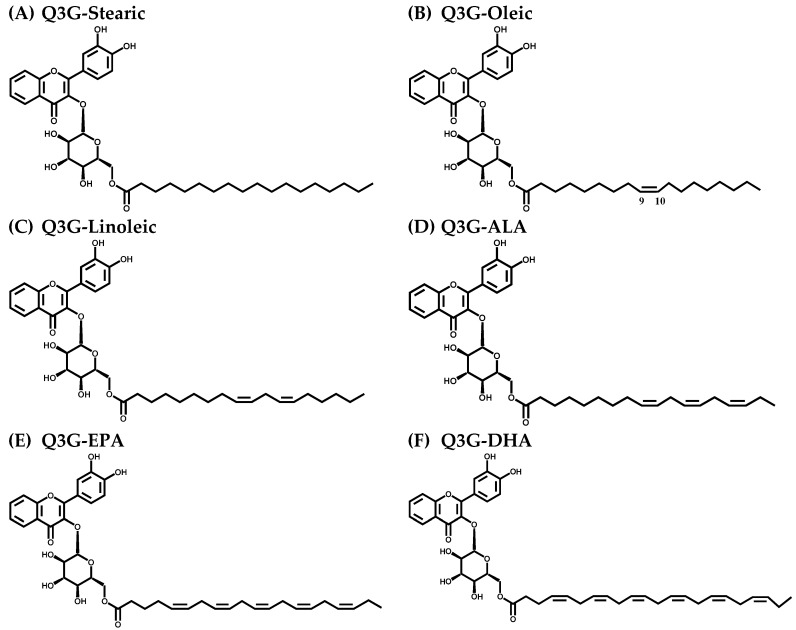
Chemical structures of six long chain fatty acid esters of quercetin-3-*O*-glucoside (Q3G). (**A**) Stearic acid ester of Q3G; (**B**) Oleic acid ester of Q3G; (**C**) Linoleic acid ester of Q3G; (**D**) α-linolenic acid (ALA) ester of Q3G; (**E**) eicosapentaenoic acid (EPA) ester of Q3G; (**F**) docosahexaenoic acids (DHA) ester of Q3G.

The quercetin molecule is lipophilic in nature, despite the number of hydroxyl groups present. However, its derivatives possess different degrees of lipophilicity depending on the type of functional groups attached to the quercetin molecule and glycosylation is known to increase the hydrophilic character [13]. In plants, glycosylation is an important modification as it makes the quercetin molecules more cytosol-soluble and also facilitates transport to different plant parts and is stored in vacuoles [14]. It was hypothesised that the acylation of the quercetin-3-*O*-glucoside (Q3G) molecule improves its lipophilicity resulting in enhanced cellular uptake and modified biological activity upon dietary intake. This study investigated the cytoprotective activity of six long chain fatty acid esters of Q3G (Figure 1) by determining the % cell viability, release of lactate dehydrogenase (LDH), production of lipid hydroperoxide and apoptotic/necrotic cell death under the H_2_O_2_-induced oxidative stress in cell culture model systems.

## 2. Results

### 2.1. Cytoprotective Effect of Fatty Acid Esters of Q3G

**Figure 2:**
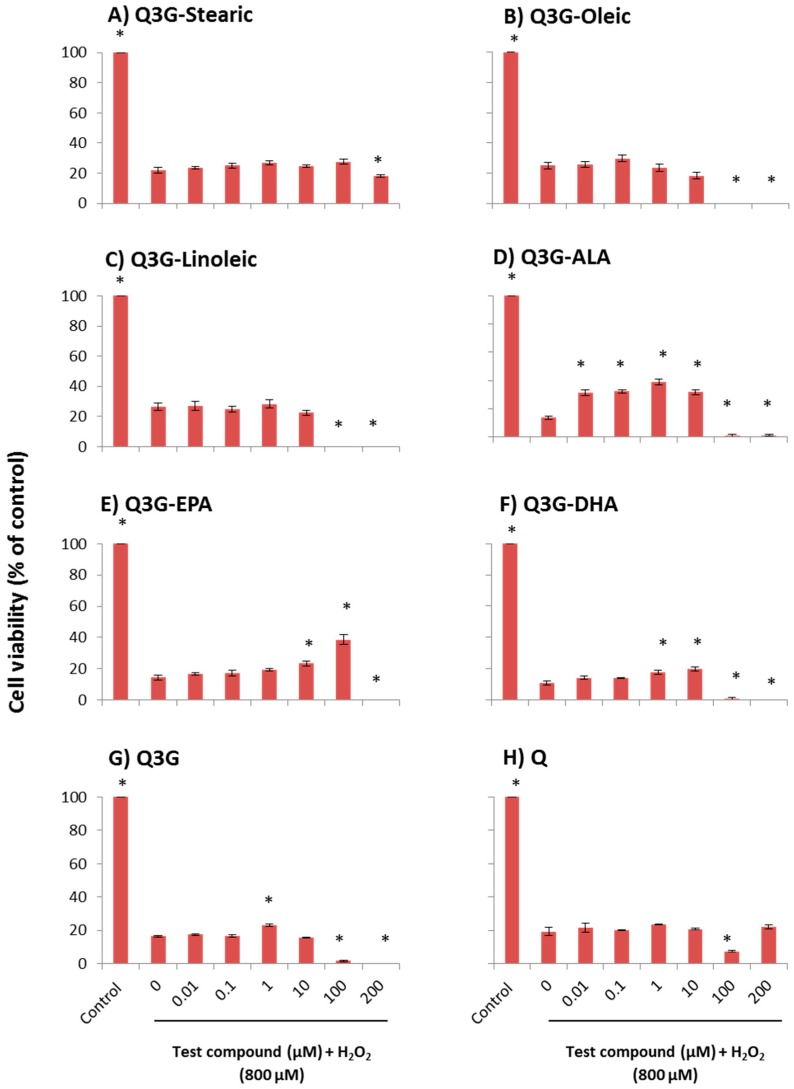
Dose-dependent cytoprotective effect of test compounds against H_2_O_2_-induced cytotoxicity in WI-38 cells. (**A**) Stearic acid ester of Q3G; (**B**) Oleic acid ester of Q3G; (**C**) Linoleic acid ester of Q3G, (**D**) ALA ester of Q3G; (**E**) EPA ester of Q3G; (**F**) DHA ester of Q3G; (**G**) Q3G and (**H**) Quercetin (Q). Cells were pre-incubated for 48 h followed by incubation with 800 µM H_2_O_2_ for 3 h. Cell viability is presented as percentage related to the control. Control contains cells with no incubation of test compounds and no oxidative injury. Data are expressed as mean ± SEM (*n* = 6). * *p* ≤ 0.05, significantly different from model group. The cells in the model group were subjected to oxidative injury by H_2_O_2_, but not treated with any test compounds.

**Figure 3:**
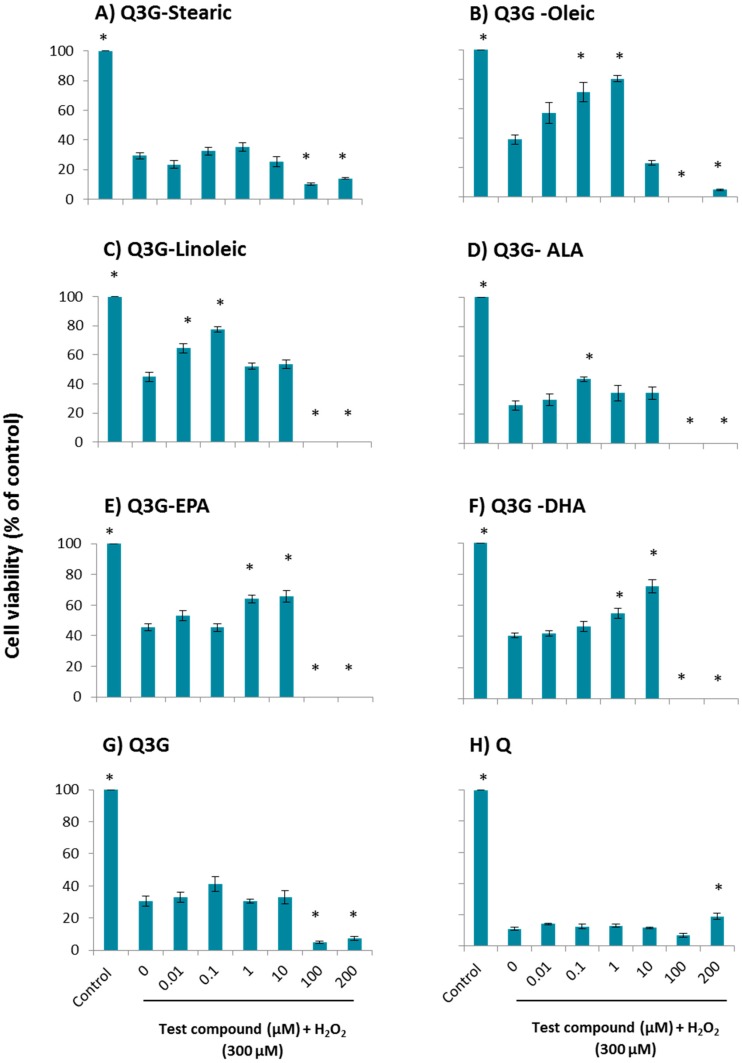
Dose-dependent cytoprotective effect of test compounds against H_2_O_2_-induced cytotoxicity in human primary hepatocytes. (**A**) Stearic acid ester of Q3G; (**B**) Oleic acid ester of Q3G; (**C**) Linoleic acid ester of Q3G; (**D**) ALA ester of Q3G; (**E**) EPA ester of Q3G; (**F**) DHA ester of Q3G; (**G**) Q3G and (**H**) Quercetin. Cells were pre-incubated for 48 h followed by incubation with 300 µM H_2_O_2_ for 3 h. Cell viability is presented as percentage related to the control. Control contains cells with no incubation of test compounds and no oxidative injury. Data are expressed as mean ± SEM (*n* = 6). * *p* ≤ 0.05, significantly different from model group. The cells in model group were subjected to oxidative injury by H_2_O_2_, but not treated with any test compounds.

The ability of the fatty acid esters of Q3G to overcome the H_2_O_2_-induced oxidative stress was determined in a dose dependent manner using normal diploid human fetal lung fibroblast cell line (WI-38) cells (Figure 2) and human primary hepatocytes (Figure 3). All viability percentages were calculated based on the untreated control with no oxidative injury. In WI-38 cells, 0.01, 0.1, 1 and 10 µM of ALA ester of Q3G, 10 and 100 µM of EPA ester of Q3G and, 1 and 10 µM of DHA ester of Q3G provided a significant (*p* ≤ 0.05) protection against acute oxidative damage, when compared to the model group of H_2_O_2_ insult without pre-incubation of any test compounds. ALA ester of Q3G demonstrated a significant cytoprotection over a wide range of concentrations compared to other compounds. It exhibited cytoprotection of 18%, 19%, 26% and 18% at 0.01, 0.1, 1 and 10 µM, respectively. Further, it is noticeable that ALA ester showed a significant protection at 0.01 µM which was a 100 times lower concentration than DHA ester and 1000 times lower than EPA ester. The concentration of 1 µM was the most effective doses of the ALA ester while for the EPA ester, 10 µM and 100 µM concentrations showed 8% and 23% protection respectively and for DHA derivative, 1 µM and 10 µM concentrations showed 7% and 9% protection, respectively. Stearic acid, oleic acid and linoleic acid esters of Q3G did not provide significant (*p* ≤ 0.05) cytoprotection at any of the tested concentrations. Both 100 and 200 µM concentrations of oleic acid, linoleic acid, ALA, and DHA esters of Q3G resulted in almost complete cell death, indicating a toxic effect at higher concentrations. Further, EPA ester also showed the same effect for 200 µM concentration. The parent compound, Q3G showed 7% cytoprotection in 1 µM concentration while it also resulted in complete loss of cell viability at 100 and 200 µM concentrations. The aglycone of Q3G, quercetin did not provide significantly higher (*p* ≤ 0.05) cytoprotection at any of the tested concentrations. The same experiment was carried out using the human primary hepatocytes (Figure 3). The long chain fatty acid esters of Q3G: 0.1 and 1 µM of oleic acid, 0.01 and 0.1 µM of linoleic acid, 0.1 µM of ALA, 1 and 10 µM of EPA and 10 µM of DHA exhibited significant (*p* ≤ 0.05) protection compared to the model group. However, stearic acid ester of Q3G did not show a significant (*p* ≤ 0.05) cytoprotection at any of the tested concentrations. The oleic acid ester provided 18%, 32% and 41% cytoprotection in 0.01, 0.1 and 1 µM, respectively. The linoleic acid ester demonstrated 20% and 33% cytoprotection at 0.01 and 0.1 µM concentrations, respectively. The cytoprotection percentages: for ALA derivative was 15% at 0.1 µM, for EPA derivative was 19% and 20% at 1 and 10 µM, for DHA derivative was 14% and 32% protection at 1 and 10 µM. All the fatty acid derivatives, except stearic acid, exhibited complete cell death at 100 and 200 µM concentrations. The parent compound, Q3G showed 11% protection at 0.1 µM concentration and 100 and 200 µM concentrations showed very low cell viability of 4%–7%. Quercetin did not give a significant (*p* ≤ 0.05) cytoprotection at any of the tested concentrations.

### 2.2. Cytotoxic Effect of Fatty Acid Esters of Q3G

**Figure 4:**
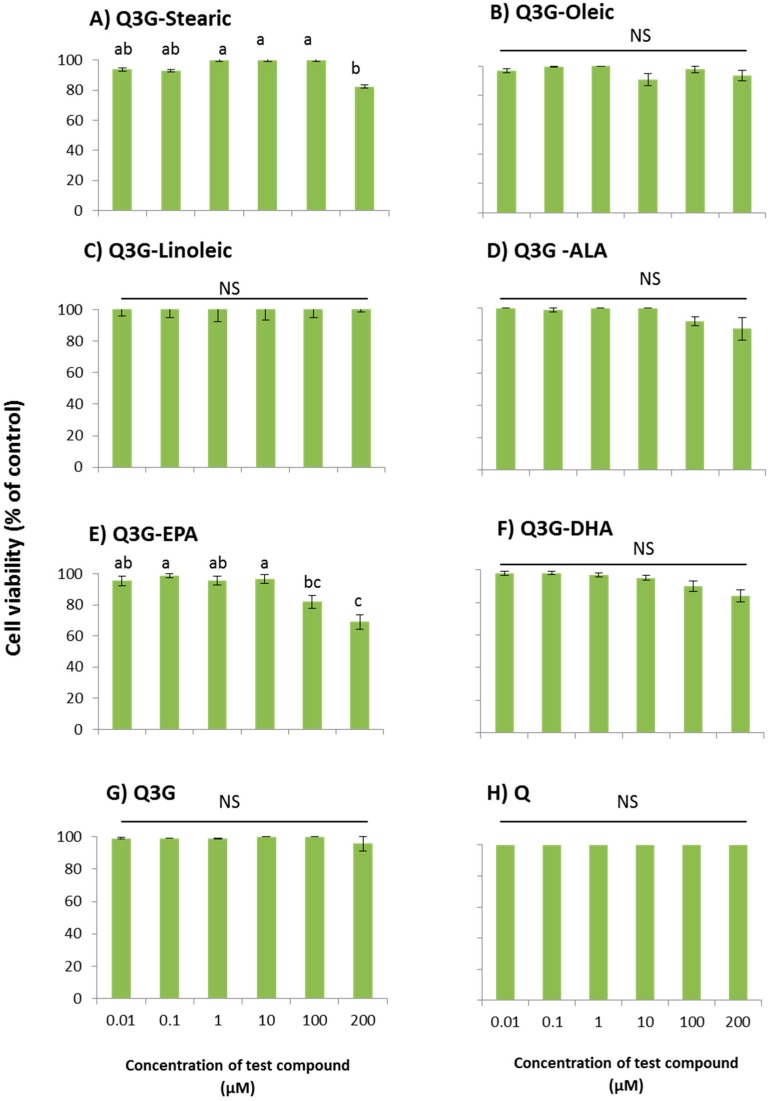
Dose-dependent cytotoxicity of fatty acid esters of Q3G in WI-38 cells; (**A**) Stearic acid ester of Q3G; (**B**) Oleic acid ester of Q3G; (**C**) Linoleic acid ester of Q3G; (**D**) ALA ester of Q3G; (**E**) EPA ester of Q3G; (**F**) DHA ester of Q3G; (**G**) Q3G and (**H**) Quercetin. Cells were pre-incubated with test compounds for 48 h. Cell viability was presented as percentage related to the control. Control contains cells with no incubation of test compounds. Data are expressed as mean ± SEM (*n* = 6), means sharing the same letter within test compound are not significantly different (*p* ≤ 0.05).

All the fatty acid esters of Q3G were tested for their toxicity for WI-38 cells in a dose dependent manner (Figure 4). Oleic, linoleic and ALA esters of Q3G were not toxic to cells at all of the tested concentrations ranging from 0.01 to 200 µM. Decrease in cell viability was observed for stearic acid ester (20% decrease at 200 µM), EPA ester (18% decrease at 100 µM and 30% decrease at 200 µM), DHA ester (10% decrease at 100 µM and 20% decrease at 200 µM) derivative of Q3G. Quercetin and Q3G did not show cell toxicity at any of the tested concentrations.

### 2.3. Cytotoxic Effect of Acyl Donor Free Fatty Acids

**Figure 5:**
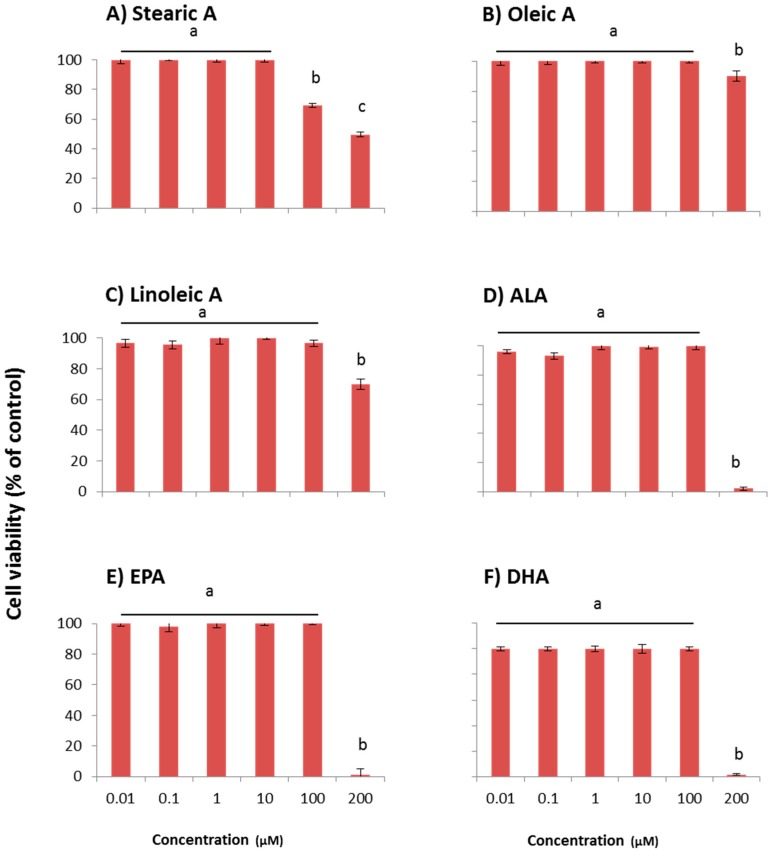
Dose-dependent cytotoxic effect of free fatty acids in WI-38 cells; (**A**) Stearic acid; (**B**) Oleic acid; (**C**) Linoleic acid; (**D**) ALA; (**E**) EPA; (**F**) DHA. Cells were pre-incubated with free fatty acids for 48 h. Cell viability was presented as percentage related to the control. Control contains cells with no incubation of free fatty acids. Data are expressed as mean ± SEM (*n* = 6), means sharing the same letter within compound are not significantly different (*p* ≤ 0.05).

The six long chain fatty acids used for acylation of Q3G were tested for their cytotoxicity against WI-38 cells in a dose dependent manner (Figure 5). With the exception of stearic acid, all other fatty acids showed no toxicity to the cells from 0.01 to 100 µM concentration range. Stearic acid reported only 69% viability at 100 µM concentration. However, all six fatty acids exhibited reduced cell viability at the 200 µM level. The cell viabilities at 200 µM were; 50% for stearic acid, 85% for oleic acid and 70% for linoleic acid. ALA, EPA, and DHA showed 100% cell death at 200 µM.

### 2.4. Cytoprotection by Fatty Acid Esters of Q3G Measured by LDH Release Assay

**Figure 6:**
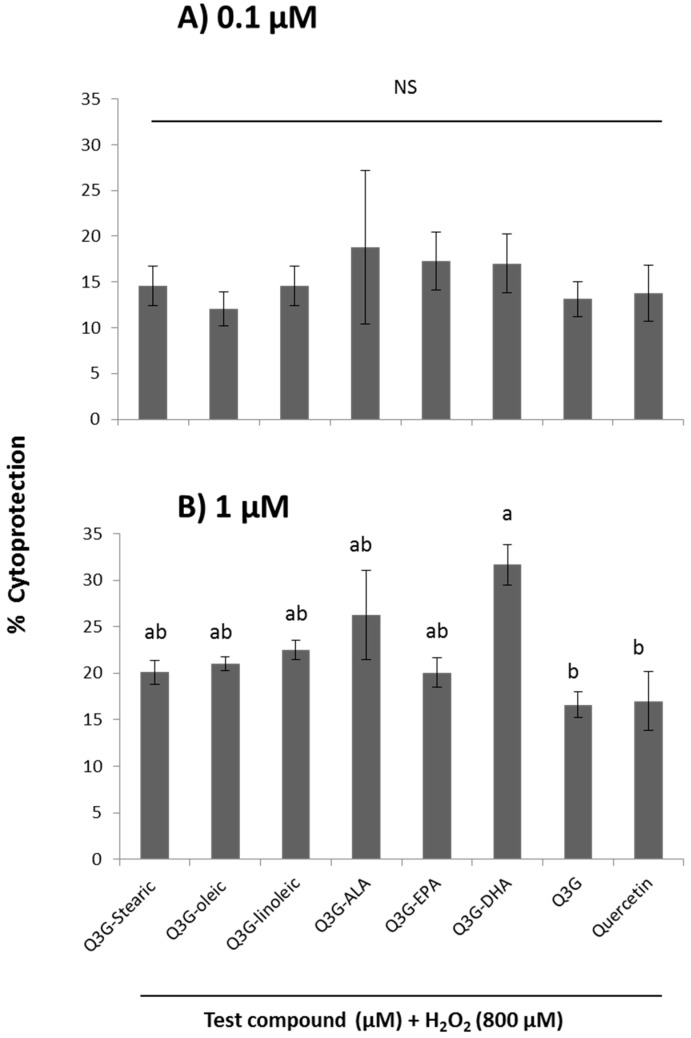
Dose-dependent cytoprotective effect of six fatty acid esters of Q3G, Q3G and quercetin using release of LDH under H_2_O_2_-induced oxidative injury in WI-38 cells. Cells were pre-incubated with test compounds at 0.1 µM (**A**) and 1 µM (**B**) followed by oxidative injury with H_2_O_2_ for 3 h. Data were expressed as mean ± SEM (*n* = 3), means sharing the same letter within concentration are not significantly different (*p* ≤ 0.05).

The cytoprotection of fatty acid derivatives of Q3G was also tested using an LDH release assay. According to the statistical analysis, the interaction effect of concentration and type of compound was not significant at *p* ≤ 0.05. However, 1 µM concentration provided significant protection over the 0.1 µM concentration level. The cytoprotection percentages exhibited by fatty acid derivatives (stearic acid, oleic acid, linoleic acid, ALA, EPA, DHA) of Q3G at 1 µM were in the range of 20%–31%. At the same concentration, the cytoprotection values for quercetin and Q3G were 16% and 17%, respectively. According to these results, the DHA derivative of Q3G demonstrated the highest protection and all the esters were able to provide higher protection than parent flavonoid Q3G and its aglycone, quercetin (Figure 6).

### 2.5. Lipid Peroxidation Under H_2_O_2_-Induced Cytotoxicity in WI-38 Cells

**Figure 7:**
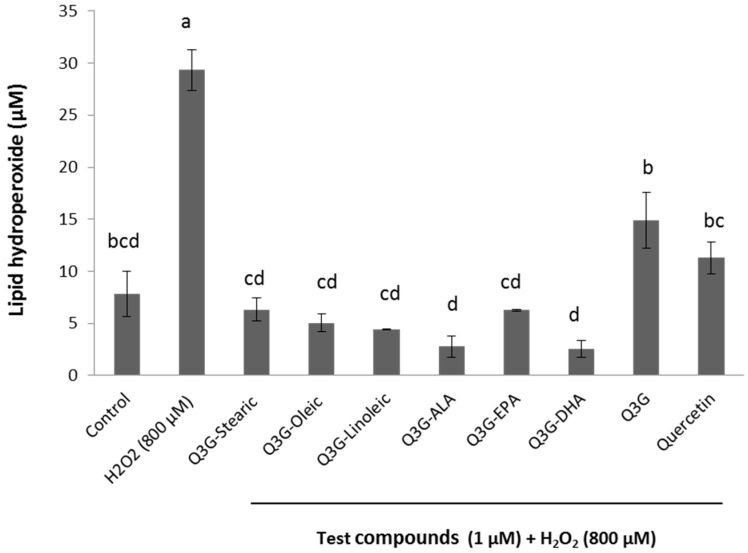
Production of lipid hydroperoxides under H_2_O_2_-induced oxidative injury in WI-38 cells. Cells were pre-incubated with test compounds for 48 h followed by 800 µM H_2_O_2_ for 3 h and production of lipid hydroperoxides was determined. Control contains the cells with no incubation of test compounds and no oxidative injury. Data were expressed as mean ± SEM (*n* = 3), means sharing same letters are not significantly different (*p* ≤ 0.05).

All the fatty acid esters of Q3G at 1 µM concentration, showed a significantly (*p* ≤ 0.05) lower production of lipid hydroperoxides compared to the H_2_O_2_ injury control (Figure 7). The decrease in lipid peroxidation by the esters was in a range of 22%–27% and ALA and DHA ester showed the highest effectiveness in inhibiting the cellular lipid peroxidation. Q3G and quercetin showed 15% and 18% decrease in the production of lipid hydroperoxides, respectively.

### 2.6. Morphological Changes and Cell Death

**Figure 8:**
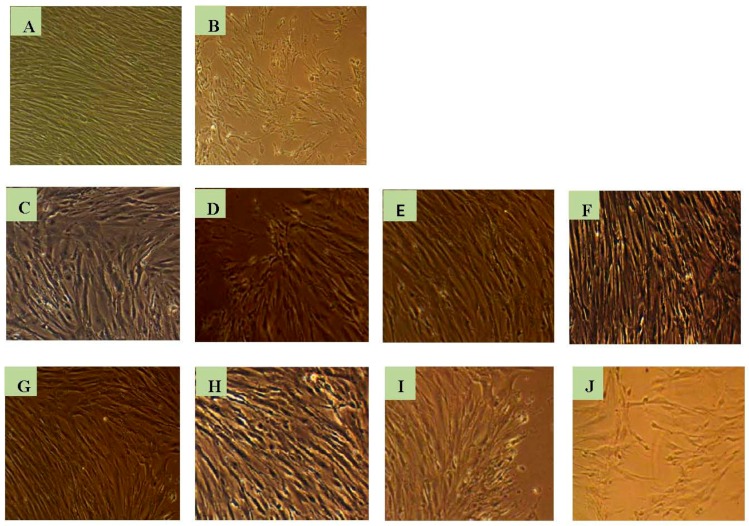
Morphological changes of WI-38 cells under an inverted phase contrast microscope (10×). (**A**) Control with no treatments; (**B**) Cells incubated only with H_2_O_2_ (800 µM) for 3 h. Images C to J represent the cells pre-incubated with test compounds for 48 h followed by H_2_O_2_-induced oxidative injury: (**C**) Stearic acid derivative of Q3G; (**D**) Oleic acid derivative of Q3G; (**E**) Linoleic acid derivative of Q3G; (**F**) ALA derivative of Q3G; (**G**) EPA derivative of Q3G; (**H**) DHA derivative of Q3G; (**I**) Q3G and (**J**) Quercetin.

The morphological changes of WI-38 cells subjected to H_2_O_2_-induced oxidative stress with and without addition of test compounds are shown in Figure 8.

**Figure 9:**
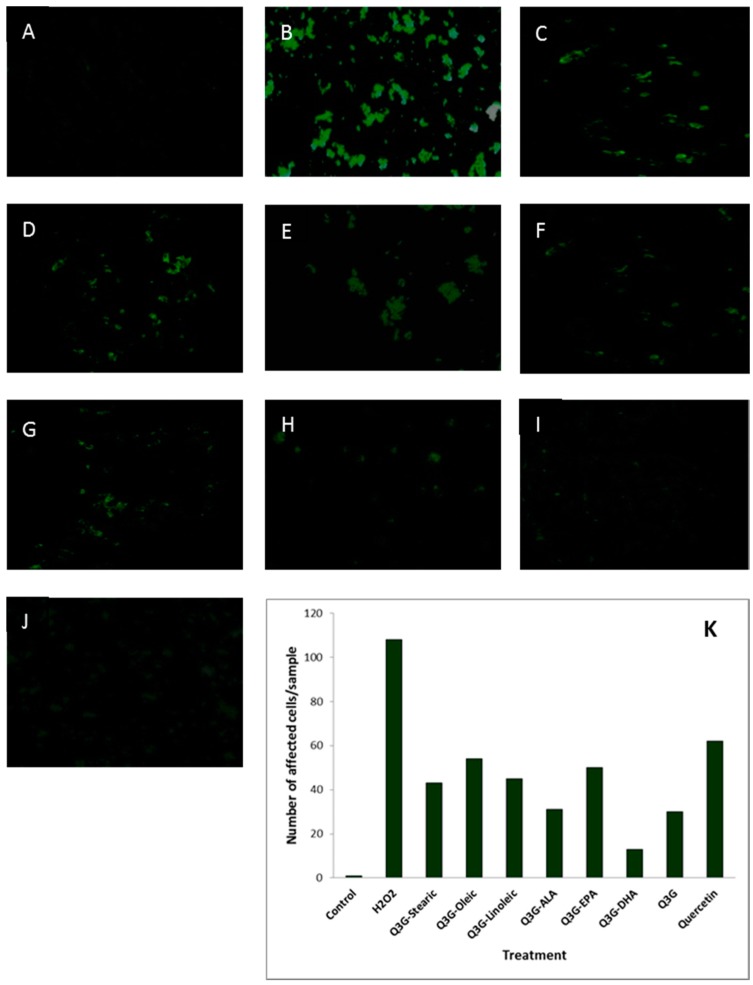
Effect of test compounds on H_2_O_2_-induced apoptotic cell death of WI-38 cells. (**A**) Untreated control; (**B**) Cells treated only with H_2_O_2_; (**C**) Stearic acid ester of Q3G, (**D**) Oleic acid ester of Q3G; (**E**) Linoleic acid ester of Q3G; (**F**) ALA ester of Q3G; (**G**) EPA ester of Q3G; (**H**) DHA ester of Q3G; (**I**) Q3G; (**J**) Quercetin; (**K**) Number of apoptotic cells in relation to the treatments. Cells were pre-incubated with test compounds for 48 h and then subjected to oxidative injury by 3 h incubation with H_2_O_2_. Cells were rinsed with PBS, stained with Annexin V and observed under a fluorescence microscope.

**Figure 10:**
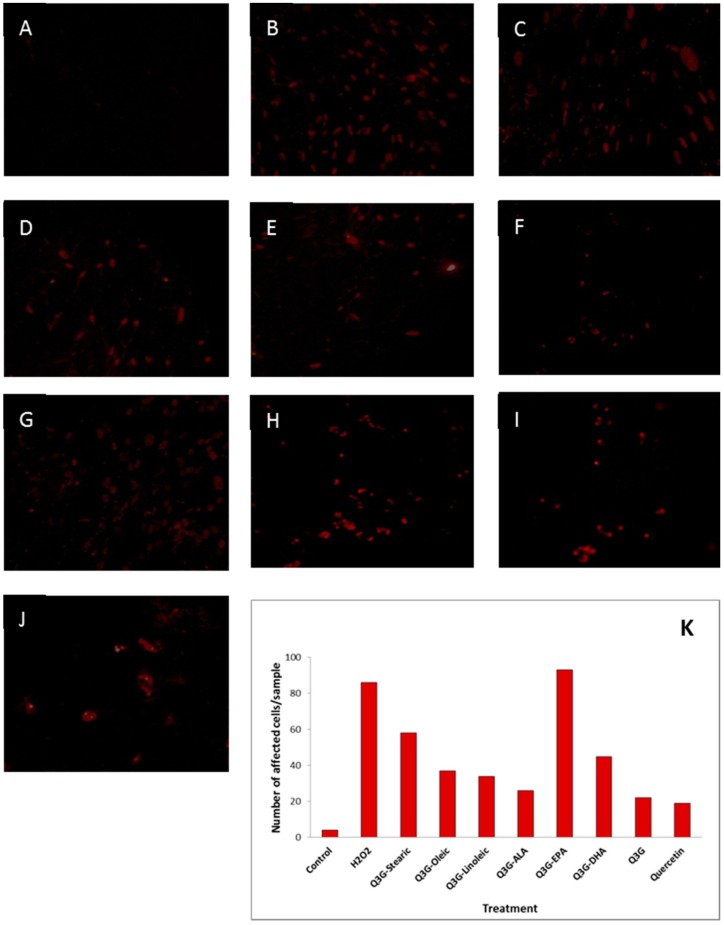
Effect of test compounds on H_2_O_2_-induced late apoptotic/necrotic cell death of WI-38 cells. (**A**) Untreated control; (**B**) Cells treated only with H_2_O_2_; (**C**) Stearic acid ester of Q3G; (**D**) Oleic acid ester of Q3G; (**E**) Linoleic acid ester of Q3G; (**F**) ALA ester of Q3G; (**G**) EPA ester of Q3G; (**H**) DHA ester of Q3G; (**I**) Q3G; (**J**) Quercetin; (**K**) Number of late apoptotic/necrotic cells in relation to the treatments. Cells were pre-incubated with test compounds for 48 h and then oxidative injured by 3 h incubation with H_2_O_2_. Cells were rinsed with PBS, stained with necrosis detection reagent and observed under a fluorescence microscope.

The morphology of the cells appeared to be altered with the incubation of H_2_O_2_ when compared to the healthy cells. The cells treated with the test compounds seemed to be less damaged, shrinked and ruptured compared to the cells which were oxidatively injured without adding the test compounds. The cells underwent pre- and late- apoptosis as presented in Figure 9 and Figure 10, respectively. Staining with fluorescence staining dyes, the early apoptotic cells were colored green and late apoptotic and/or necrotic cells were colored red. The cells pre-treated with test compounds showed less stained area compared to the oxidatively damaged cells, but not treated with test compounds.

## 3. Discussion

Plant-based polyphenolic antioxidants have been extensively investigated for their potential therapeutic applications against oxidative stress associated pathogenesis of chronic diseases [15]. The focus of the current study was directed on cytoprotection properties of six novel long chain fatty acid esters of a flavonoid, Q3G, using cell model systems of oxidative stress. The cytoprotective effect of flavonoids and flavonoid-rich extracts under induced oxidative stress conditions in different cell culture model systems are well documented [9,12,16,17]. A culture of hepatocytes preserves the metabolic enzymes in the liver *in vivo* providing an *in vitro* model for preliminary screening on hepatoprotective activity of test compounds. Among them, primary hepatocytes are considered to be the closest to the liver *in vivo* [6,18]. Hesperidin or hesperetin-7-*O*-rutinoside [11], a flavonoid extracted from *Artemisia capillaries* L. [19] and an extract of bilberry [20] are reported to have protective effects against *tert*-butylhydroperoxide-induced oxidative stress in human hepatocytes and rat primary hepatocytes. Moreover, as a highly exposed organ for high oxygen concentration, the lungs are subjected to many airway diseases and the role of human lung fibroblasts is imperative in pulmonary hypertension, pulmonary fibrosis, diffuse alveolar damage by thickening and/or remodeling of airways and parenchyma [21]. Cytoprotective effects of a flavonol, morin [10], hyperoside (quercetin-3-*O*-galactoside) [9] and modified curcumin [16] have been reported against H_2_O_2_-induced cell damage of Chinese hamster lung fibroblasts [10].

The lipophilic/hydrophilic nature of the antioxidants plays a significant role in determining their biological activity as a crucial factor governing the membrane permeability and target accessibility [22]. Therefore, conjugation of hydrophobic moiety to the antioxidant molecule is practiced widely and it has been shown that hydrophobic groups contribute highly to the cellular uptake and biological activity of phenolic compounds [23]. However, the dispute on the role of lipophilicity in enhancing the antioxidant activity still remains unanswered. Recently, it has been reported that lipophilicity acts as a double-edge sword due to the non-linear relationship between lipophilicity and antioxidant activity [24].

To our knowledge, studies on the effect of increased lipophilicity to mitigate the oxidative stress-induced cytotoxicity are still very limited. Caffeic acid esters with higher lipophilicity exhibited dose-dependent cytoprotection against oxidative damage in rat pheochromocytoma cells [22]. Also, stearic acid, EPA and DHA esters of epigallocatechin gallate (EGCG) were more effective than EGCG, in protecting against DNA scission induced by hydroxyl and peroxyl radicals [25]. Similarly, acylation of phloridzin and Q3G with long chain fatty acids provided anti-proliferative properties to the polyphenol compound as tested hepatocellular carcinoma HepG2 cells [26,27]. Acylation of flavonoids also provided enhanced anti-inflammatory function in lipopolysaccharide-induced inflammation in THP-1 differentiated macrophages [28,29]. Antioxidant activity of chlorogenic acid and its methyl, butyl, octyl, dodecyl and hexadecyl esters towards mitochondrial ROS generated in a ROS-overexpressing fibroblast cell line was investigated and long chain esters were found to be more effective in scavenging ROS [30]. Abilities of flavonoids to prevent the formation of DNA damages in cells exposed to H_2_O_2_ was positive in Jurkat cells (an immortalized line of human T lymphocyte cells) and it has been proposed that cytoprotective effect of flavonoids depends on their ability to penetrate through the plasma membrane and to remove loosely bound redox-active iron from specific intracellular locations [31].

H_2_O_2_ and *tert*-butylhydroperoxide are commonly used in cell culture studies to mimic oxidative stress *in vitro*. H_2_O_2_ is a major component of intracellular ROS generated during normal physiological processes and elevated levels are reported under pathological conditions [10,32]. Due to its less toxic nature and diffusion through biological membranes to reach the target sites, H_2_O_2_ has been widely used as an inducer of oxidative stress *in vitro* [4,33]. Therefore, in the present study, H_2_O_2_ was used to induce oxidative stress in two cell culture systems. In this study, the fatty acid esters of Q3G were better antioxidants than Q3G and quercetin for attenuating the cytotoxicity induced by H_2_O_2_ in both primary hepatocytes and lung fibroblasts. However, an aggravated effect was observed at 100 or 200 µM of the test compounds indicating the possible pro-oxidant activity at higher doses under oxidative injury. Quercetin is sparingly soluble in the cell culture medium at higher concentration [34] and this could be the reason for no cytotoxicity observed at 200 µM for quercetin under induced oxidative stress while it was toxic at 100 µM. There was no cytoprotective effect demonstrated by Q3G against oxidative stress in rat C2 glioma cells while it was not toxic at 100 µM [35]. Our findings in current study are similar. The most probable reason for the cytoprotective effect of Q3G esters over Q3G or quercetin is their improved lipophilic nature which provides permeability through cell membranes. However, the toxic effect observed at the higher concentrations is possibly due to several reasons. Firstly, the high intracellular concentrations or interactions with cell membranes could generate pro-oxidative effects. Secondly, the aggregative effect of Q3G and the toxicity of the acyl donor fatty acids at higher doses provide important evidence. The cytotoxicity of fatty acids has been discussed in the literature focusing the effect of structural diversity in carbon chain. Fatty acid-induced apoptosis and necrosis were exhibited in macrophages while no clear relationship was observed between the toxicity and carbon chain length or the number of double bonds [34]. However, arachidonic acid, linolenic acid, linoleic acid and oleic acid were reported to be toxic in human lens epithelial cells and it has been further found that saturated fatty acids were less effective than the unsaturated fatty acids [36]. However, post treatment with the test compounds was not able to demonstrate any cytoprotection in either cell system (data not shown). The acute cytotoxicity created in the experiment could be too harsh for the cellular system to become regenerated with the activity of test compounds.

Lipid peroxidation is considered as the major mechanism of cell injury caused by H_2_O_2_ [10,18] and therefore, it is a reliable indicator of the cellular oxidative damage [37]. Metal ions are capable of catalyzing the cytotoxic effect of H_2_O_2_ and therefore polyphenols effective in chelating the metal ions are protectors against H_2_O_2_-induced cytotoxicity [38]. Oxidative stress can induce the cell death via either apoptosis or necrosis and the degree of the oxidative insult determines the pathway triggered [39]. A previous study demonstrated that H_2_O_2_ triggered the apoptosis pathway at 10 and 100 µM on human lung fibroblast cells and DNA strand breaks were observed in terminal deoxynucleotidyl transferase dUTP nick end labeling (TUNEL) assay [21]. Further, necrotic cell death was observed at H_2_O_2_ concentrations higher than 1 mM.

In general, antioxidant activity depends on the number, location and substitution pattern of hydroxyl groups in flavonoids [40,41] which scavenge free radicals generating less reactive phenoxyl radicals or chelate transition metal ions suppressing the Fenton reaction [9]. Chelating property of flavonoids depends mainly on the catechol group on the B ring, 4-carbonyl, and 5-hydroxyl group [9]. Further, the amount of flavonol taken up by the cells and consequently, its cytoprotection effect is mastered by the lipophilicity of the structure [12]. Kale (*Brassica oleracea* L.) extract failed to protect and even aggravated the deleterious effects of H_2_O_2_-induced acute oxidative stress in Chinese hamster lung fibroblast cells [38]. However, kaemferol-3-*O*-rutinoside, ferulic acid and sinapic acid didn’t prevent nor aggravate the toxicity induced by H_2_O_2_ in V79 cells. It has been postulated that the higher molecular weight and the polar nature of those molecules rather than their aglycones hinder their movements across the cell membrane [5]. However, rutin (quercetin-3-*O*-rutinoside) attenuates H_2_O_2_-induced cytotoxicity and apoptosis in human umbilical vein endothelium cells (HUVEC) via ROS down-regulation, reduced glutathione (GSH) up-regulation and restoration of mitochondrial membrane potential [17]. In cell culture models, cytoprotective effects of flavonoids are highly linked to their antioxidant activity. Furthermore, lipophilicity of the flavonoid molecule facilitates the passing through the bilayer in the cell membranes. Therefore, lipophilic character together with supportive chemical structure has a significant role in the potency of optimum cytoprotective effects [42].

It is quite clear that the antioxidant activities of the naturally-occurring and preferentially absorbed quercetin derivatives are very different to quercetin aglycone [43]; however, the natural phytochemical form is not necessarily the bioactive form *in vivo* [44]. Biotransformation of flavonoid molecules take place in intestine and liver and their absorbed metabolites are crucial for bioavailability [45]. Naturally occurring flavonoids are found mainly in their glycosylated form in which the position and number of glycosylation are responsible for its absorption and bioactivity in human body [46]. During the initial phase in flavonoid metabolism, deglycosylation process is known to take place and the resulting aglycones are metabolized into glucuronides, sulphates and *O*-methylated forms [47,48]. The effects of these metabolites at the cellular level depend on their interactions with the cell membranes and the uptake into the cytosol which can vary according to the cell type. Furthermore, their potential as mediators in cellular signal transduction is gaining an importance. However, the true bioactive form of the flavonoids is still under debate. This raises the need of better understanding the bioavailability of flavonoids, their availability for absorption (bio-accessibility), tissue distribution and bioactivity [49], using animal studies.

## 4. Materials and Methods

### 4.1. Cell Cultures

Normal diploid human fetal lung fibroblast cell line (WI-38) was obtained from American Type Culture Collection (ATCC, Manassas, VA, USA). The cells were grown in ATCC formulated Eagle’s minimum essential medium (EMEM) supplemented with fetal bovine serum (FBS, ATCC) to a final concentration of 10% at 37 °C in a 5% CO_2_ and humidified environment (CO_2_ incubator, Model 3074, VWR International, West Chester, PA, USA). Cells were maintained in culture up to 40 population doublings in T-75 flasks (Becton Dickinson Labware, Bedford, OH, USA).

Fresh human normal primary hepatocytes (h-NHEPS™) were purchased from Lonza (Walkersville, MD, USA). The cells were cultured in hepatocyte basal medium (HBM™) supplemented with the HCM™ [ascorbic acid, bovine serum albumin-fatty acid free (BSA-FAF), hydrocortisone, transferrin, insulin, recombinant human epidermal growth factor (rhEGF), GA-1000 (gentamicin, amphotericin-B)] at 37 °C in a 5% CO_2_ and humidified environment. No sub-culturing was recommended by the manufacturer as cells undergo replicative senescence.

### 4.2. Synthesis of Fatty Acid Esters of Q3G

Six fatty acids, namely stearic acid, oleic acid, linoleic acid, linolenic acid (ALA), eicosapentaenoic acid (EPA) and docosahexaenoic acid (DHA) derivatives of Q3G were enzymatically synthesized according to the method described in [50]. Briefly, 500 mg of Q3G (Indofine Chemical Company, Hillsborough, NJ, USA) and each acyl donor fatty acids (Nu-Check prep Inc., Elysian, MN, USA) were reacted in a molar ratio of Q3G:acyl donor 1:5 using anhydrous acetone as the solvent. The acylation was initiated by adding Novozym 435^®^ immobilized lipase from *Candida antarctica* L. (2 g) as the biocatalyst followed by an incubation at 45 °C for approximately 48 h. After the completion of the reaction, the product was isolated by silica gel column chromatography using acetone–toluene, 40:60 to 50:50 elution monitored by preparative thin layer chromatography (TLC).

### 4.3. MTS Assay

Cells were cultured in 96-well plates at a density of 1 × 10^4^ cells/100 µL per well and incubated at 37 °C for 24 h to allow for the attachment of cells. The cells were then treated with 100 µL test compounds (0.01, 0.1, 1, 10, 100 and 200 µM) in DMSO (<1%, *v*/*v*) and incubated at 37 ^°^C for 48 h. The cells in control group contained equal volume of DMSO without adding any test compounds and were not subjected to oxidative injury. The cells in model group contained equal volume of DMSO, no addition of test compounds, but were oxidatively injured by H_2_O_2_ addition. Exposure time and concentration of H_2_O_2_ were optimized (Appendix A). After incubation, culture medium was removed and washed with PBS twice, applying centrifuging at 1000 rpm for 2 min in between. Then, cells were subjected to H_2_O_2_ insult at 800 µM for lung fibroblasts and 300 µM for normal hepatocytes followed by 3 h incubation at 37 °C. H_2_O_2_ was freshly prepared each time in serum free media using 30% H_2_O_2_ solution (Sigma Aldrich, Mississauga, ON, Canada). Then, media was removed and cells were rinsed with PBS to remove the oxidant and 100 µL of fresh serum free media was added into the wells. CellTitier 96^®^ aqueous non-radioactive cell proliferation assay (Promega Corporation, Madison, WI, USA) was used to determine the cell viability. The absorbance was measured at 490 nm using a plate reader (FLUOstar OPTIMA, BMG Labtech, Burham, NC, USA).

$$\%\text{Cell viability}=\frac{\text{Absorbance of the treated wells}-\text{Blank}}{\text{Absorbance of the control wells}-\text{Blank}}\times 100$$

where the treated wells contained the cells, pre-incubated with test compounds followed by oxidative injury, and the control wells contained the cells with no incubation of test compounds and no oxidative injury. Blank wells contained culture media only.

### 4.4. Cytotoxicity of the Fatty Acid Acylated Esters of Q3G

WI-38 cells were cultured in 96-well plates at a density of 1 × 10^4^ cells/100 µL per well and incubated at 37 °C for 24 h. Cells were treated with 100 µL of the test compounds (0.01, 0.1, 1, 10, 100 and 200 µM) and incubated at 37 °C for 48 h. Then, the culture medium was removed and cells were washed with PBS twice. Cell viability was determined as described before.

### 4.5. Cytotoxicity Assessment of the Free Fatty Acids Used as Acyl Donors

WI-38 cells were cultured in 96-well plates at a density of 1 × 10^4^ cells/100 µL per well and incubated at 37 °C for 24 h. Then, cells were treated with 100 µL of the fatty acids separately: stearic acid, oleic acid, linoleic acid, ALA, EPA, DHA in 0.01, 0.1, 1, 10, 100, and 200 µM concentrations and incubated at 37 °C for 48 h. Cell viability was determined as described before.

### 4.6. LDH Release Assay

WI-38 cells were cultured in 96-well plates at a density of 1 × 10^3^ cells/100 µL per well and incubated at 37 °C for 24 h. According to the result from the MTS assay, 0.1 and 1 µM concentrations were selected for the test compounds. After treating the cells with 100 µL test compounds, they were incubated at 37 °C for 48 h. The cells in the control group contained equal volume of DMSO (less than 0.1%, *v*/*v*) without adding any test compounds and were not oxidatively injured. The cells in the model group contained equal volume of DMSO, no addition of test compounds, but subjected to oxidative injury by H_2_O_2_ addition. Then the culture medium was removed and cells were washed with PBS twice. Cells were subjected to H_2_O_2_ insult of 800 µM for 3 h. After incubation, the plate was centrifuged at 1000 rpm for 2 min and 50 µL of the supernatant was added into a new 96-well plate. The released LDH was assayed using CytoTox 96^®^ Non-Radioactive Cytotoxicity Assay kit (Promega Corporation) according to the manufacturer’s protocol. Absorbance was measured at 490 nm using a plate reader (FLUOstar OPTIMA). After subtracting the absorbance of blank and control wells, from all the model wells and test wells, cytoprotection was calculated as below:

$$\%\text{Cytoprotection}=\frac{\text{Absorbance of the model wells}-\text{Absorbance of the test wells}}{\text{Absorbance of the model wells}}\times 100$$

where, the model wells contained the cells, oxidative injured without pre-incubation with the test compounds and the test wells contained the cells, pre-incubated with the test compounds followed by oxidative injury. The blank contained only the culture media. The control wells contained the cells with no incubation of test compounds and no oxidative injury.

### 4.7. Lipid Peroxidation Assay

Lipid peroxidation was measured using a lipid hydroperoxide assay kit (Cayman Chemical Company, Ann Arbor, MI, USA). WI-38 cells were cultured in 24-well plates at a density of 1 × 10^5^ cells/mL per well and incubated at 37 °C for 24 h. Then, the cells were treated with 1 µM of test compounds for 48 h at 37 °C. After incubation, the cells were washed with PBS to remove the test compounds and incubated with 800 µM H_2_O_2_ for 3 h at 37 °C. At the end of the oxidative injury period, supernatant (200 µL) of each well was taken into glass vials and lipid hydroperoxides were extracted by adding the equal volume of Extract R saturated methanol and 1 mL of cold chloroform followed by centrifuging the pre-vortexed mixture at 1500× *g* for 5 min at 0 °C. The bottom chloroform layer (500 µL) was collected to a glass vial and stored on ice. Methanol and chloroform was deoxygenated prior to use by bubbling nitrogen through the solvents for about 30 min. Deoxygenated chloroform–methanol (2:1) mixture (450 µL) was added into each extracted sample of 500 µL. Then freshly prepared standard chromogen (50 µL) was added into the assay vials and vortexed. The assay vials were closed tightly and kept at room temperature for 5 min. A volume of 300 µL from each vial was transferred into a glass 96-well plate and absorbance was read at 490 nm using a plate reader (FLUOstar OPTIMA). Lipid hydroperoxides in the samples were calculated using the equation obtained from the linear regression of the standard curve prepared using 0, 0.5, 1.0, 2.0, 3.0 and 4.0 nmol of lipid hydroperoxide standards.

### 4.8. Fluorescence Microscopy Assay

WI-38 cells were cultured in chamber slides (Nunc Lab-Tek II Chamber Slide System, Thermo Fisher Scientific, Ottawa, ON, Canada) at a density of 1 × 10^5^ cells/ml per well and incubated at 37 °C for 24 h. Cells were treated with 1 µM of test compounds for 48 h at 37 °C. After incubation, the cells were washed with PBS to remove the test compounds and treated with 800 µM H_2_O_2_ for 3 h at 37 °C. The cells were carefully washed with PBS twice to remove the oxidants. The dual detection reagents; apoptosis detection reagent (Annexin V-EnzoGold) and necrosis detection reagent which is similar to the red emitting dye 7-AAD, supplied by GFP-certified^™^ apoptosis/necrosis detection kit (Enzo Life Sciences International, INC., Plymouth Meeting, PA, USA) were prepared in binding buffer (1×). After careful removal of the supernatant, the prepared detection reagent was dispensed in a volume sufficient for covering the cell monolayer in each slide. The cells were incubated for 15 min at room temperature, protected from the light. Then, the staining solution was flicked onto a paper towel and few drops of binding buffer were added to prevent the cells from drying out. The cells were then observed under fluorescence microscope (DMBL 20x40 magnification, Leica Microsystems, Houston, TX, USA) with a filter sets for Cyanine-3 (Ex/Em: 550/570 nm) and 7-AAD (Ex/Em: 546/647 nm), coupled with digital camera (Nikon Cool Pix 4500, Nikon Canada Inc., Mississauga, ON, Canada). Number of affected cells were also counted for quantification of apoptosis and late apoptosis/necrosis.

### 4.9. Cell Morphological Assessment

WI-38 cells were cultured in 24-well plates at a density of 1 × 10^5^ cells/1 ml per well and incubated at 37 °C for 24 h. The cells were treated with 1 µM of test compounds for 48 h at 37 °C. After incubation, the cells were washed with PBS to remove the test compounds and treated with 800 µM H_2_O_2_ for 3 h at 37 °C. Morphology of the cells was examined under inverted microscope (ECLIPSE TS 100/TS 100-F, Nikon Instruments Inc., Melville, NY, USA) using phase contrast optics under 10× magnification. The images were recorded using the Lumenara Infinity camera (1–2 USB, 2.9 Megapixel) including capture and analyzing software (Infinity Analyze, Lumenara Corporation, Ottawa, ON, Canada).

### 4.10. Statistical Analysis

The experiments done with MTS assay were carried out in six replicates and all others in three replicates. The results were expressed as mean ± standard error mean (SEM). Statistical analysis was performed using one way ANOVA in Minitab 16 statistical software and multiple mean comparison was carried out by Tukey’s method. *p* ≤ 0.05 was considered as statistically significance. For the statistical determination of the assays conducted for cytoprotection effects and cytotoxic effects using MTS assay, compounds were analyzed individually to understand their concentration effect. For the LDH assay, the series of test compounds was analyzed statistically under two concentration levels separately. The statistical determination for the lipid peroxidation assay was performed to determine the significance effect of the different test compounds at one concentration level.

## 5. Conclusions

In conclusion, H_2_O_2_-induced cytotoxicity was attenuated by ALA, EPA and DHA acid esters of Q3G in human primary hepatocytes and lung fibroblasts as demonstrated by significantly higher % cell viability (*p* ≤ 0.05) under the experimental condition of H_2_O_2_-induced oxidative injury in cell culture model systems. Oleic acid and linoleic acid esters of Q3G exhibited significant (*p* ≤ 0.05) cytoprotection only in primary hepatocytes. ALA and DHA esters of Q3G exhibited significantly (*p* ≤ 0.05) lower production of lipid hydroperoxides under oxidative stress conditions. Low apoptotic cell death was demonstrated with the pre-incubation of cells with the novel ester compounds. Therefore, fatty acid esters of Q3G can be considered as potential cytoprotective agents under oxidative stress.

## Appendix A

**Table A1:** Effect of concentration and exposure time of H_2_O_2_ on the percentage cell viability of human primary hepatocytes.

Concentration of H_2_O_2_ (µM)	Cell Viability (% ± SD) for Primary Hepatocytes		
	1 h	3 h	6 h
100	75.12 ± 1.41	60.26 ± 1.56	15.84 ± 1.62
300	44.86 ± 1.04	25.53 ± 2.47	8.37 ± 1.38

The cell viability was measured using MTS assay as described under Section 4.3.

**Table A2:** Effect of concentration and exposure time of H_2_O_2_ on the percentage cell viability of human lung fibroblasts (WI-38 cells).

Concentration of H_2_O_2_ (µM)	Cell Viability (% ± SD) for WI-38 Cells			
	1 h	3 h	6 h	12 h
300	62.31 ± 0.26	56.02 ± 0.05	40.32 ± 1.36	19.85 ± 0.21
500	49.63 ± 1.53	39.05 ± 1.21	20.71 ± 0.37	15.45 ± 1.25
800	41.52 ± 1.46	28.23 ± 0.36	8.41 ± 1.20	0 ± 0.00

The cell viability was measured using MTS assay as described under Section 4.3.
